# Effects of Dextromethorphan on Depressive-and Cognitive-Associated Behaviors: A Sexually Dimorphic Study

**DOI:** 10.32598/bcn.9.10.275

**Published:** 2019-07-01

**Authors:** Omamuyovwi Meashack Ijomone, Ifechukwude Joachim Biose

**Affiliations:** 1. Department of Anatomical Sciences, School of Health and Health Technology, Federal University of Technology Akure, Nigeria.; 2. Department of Anatomy and Forensic Anthropology, Faculty of Basic Medical Sciences, Cross River University of Technology, Calabar, Nigeria.

**Keywords:** Dextromethorphan, Depression, Cognition, Hippocampus, Female, Male

## Abstract

**Introduction:**

We investigated the sexually dimorphic effects of Dextromethorphan (DM) on cognitive and depression-like behaviors as well as on hippocampal histology in rats following acute administration.

**Methods:**

Wistar rats of both sexes were treated with 25 or 50 mg/kg of DM for 7 days via intraperitoneal injection. At the end of the administration, behavioral studies were performed on the Tail Suspension Test (TST) for depressive-like behaviors and the Y-maze for cognitive behaviors. The rats’ brains were excised and processed for routine histological analysis.

**Results:**

Our results showed that DM significantly increased (P<0.05) immobility time in the TST in male rats but not female ones, and decreased percentage alternation (P<0.001) on the Y-maze in both male and female rats. Histological analysis revealed no morphological changes in the hippocampus following DM treatment.

**Conclusion:**

DM impairs cognitive functions in both male and female rats without histologic defects in the hippocampus. However, the induced depressive-like behaviors following DM administration may be sexually dependent.

## Highlights

- Dextromethorphan significantly increased depression-like behavior in male rats.
- Cognitive functions were significantly impaired in both male and female rats.
- Dextromethorphan did not cause morphological changes in brain histology.

## Plain Language Summary

The recreational use of Dextromethorphan (DM), a major constituent of cough-syrups is increasing globally. Also, DM is currently under investigation to be used clinically for pain management. Little is known about the impact of DM abuse on behavioral patterns such as memory and depression. We examined DM effects on behaviors using rat models and their dependency on gender. Our result demonstrates that DM impairs memory functions of the brain in both male and female rats without morphological changes in the brain. Also, DM produced significant depression in male rats. Also, we report that DM impairs cognitive behaviors in both genders. Altogether, given the differential increase in substance abuse among males and rising global reports of depression-motivated suicides, the depressive effects of DM should be considered and investigated.

## 1. Introduction

Dextromethorphan (DM) is a globally available over-the-counter substance, delivering mostly in antitussive drug formulations. DM, synthesized in the form of a white powder, is a dextro isomer of levomethorphan –a derivative of morphine. While DM bears some chemical similarities with other opiates such as morphine and heroin, it is not an opioid receptor agonist (Expert Committee on Drug Dependence, 2012). However, DM blocks N-Methyl-D-Aspartate (NMDA) receptors and is a potent sigma-1 receptor agonist (Shin et al., 2007). Hence, DM has pharmacological effects similar to those of commonly abused substances such as ketamine, psilocybin, and lysergic acid diethylamide (Banken & Foster, 2008).

The recreational use of DM is an increasing global trend, due to ease of accessibility, dissociative and addictive potentials, and the perception of safety (Pringle, McDonald, & Gabriel, 2015). DM is a well-described abused substance (Roy, Hsieh, & Crapanzano, 2015) among young adults across the world (Logan et al., 2012). Believed to be the most commonly used dissociative substance in many climes (Storck, Black, & Liddell, 2016), DM use has been categorized into three classes; suicidal, misuse, and addictive (Gibaja et al., 2016; Shafi et al., 2016). Therefore, DM is a psychotropic substance that carries the potential for abuse and addiction with mild withdrawal symptoms (Mutschler et al., 2010).

Nevertheless, many therapeutic applications and clinically relevant effects of DM are currently under investigation. One such beneficial therapeutic use of DM is its effect on acute neuropathic pain in both preclinical (Karimi, Tabrizian, & Rezaee, 2010) and clinical studies (Shaibani, Pope, Thisted, & Hepner, 2012). Reports suggest that DM, as a potent NMDA antagonist, compromise spatial learning, and memory (Zhang et al., 2007). NMDA receptors in the hippocampus are vital for learning and memory (Berg, Larsson, Morland, & Gundersen, 2013). Conversely, blocking NMDA-receptors impairs memory formation during learning activities (McHugh, Niewoehner, Rawlins, Bannerman, 2008). On the other hand, there are conflicting reports on the effects of DM on depression as studies have reported both an anti-depressant effect (Nguyen & Matsumoto, 2015) as well as depressive-like effects (Po et al., 2015).

Sexual differences are indispensable variables in biomedical research, though this has been essentially ignored. In this regard, only 10% of animal studies in neuroscience use female animal models (Beery & Zucker, 2011). It is essentially speculative to ascribe neurobehavioral findings in males to females; hence, the National Institute of Health recommends the inclusion of female rodents for preclinical research (Sandberg, Umans, & Georgetown Consensus Conference Work Group, 2015). In addition, accumulating evidence suggests dimorphic sexual effects of antidepressant drugs (Kokras & Dalla, 2014). Given this, we aimed to investigate the sexually dimorphic effects of DM on depressive-like and cognitive behaviors as well as on hippocampal histology in rats following acute administration of DM.

## 2. Methods

### 2.1. Animal care and treatment

Adult Wistar rats of both sexes (average weight: 170 g) were used in the current study. The animals had access to standard laboratory rodent chow and water and kept in clean habitat. All protocols involving rats were guided by in the NIH Guidelines for the Care and Use of Laboratory Animals and approved by the Local Institutional Research and Ethics Committee.

A total of 34 animals were used. The animals were grouped into 3 of both sexes. The control group (n=10; 5 females and 5 males) received normal saline. The animals in the two other groups received DM at 25 mg/kg (n=12; 6 males and 6 females) or 50 mg/kg (n=12; 6 males and 6 females). DM was administered as dextromethorphan hydrobromide (Long-Range Europe Ltd, UK). All administrations were via intraperitoneal injection for 7 days. DM dose selection was based on a previously published study by Carliss et al., (2007). At the end of the administration, tail suspension and Y-maze were performed for depressive and cognitive associated behaviors, respectively. After the neurobehavioral tests, the rats were euthanized via cervical dislocation. Their brains were quickly removed and fixed in Neutral Buffered Formalin (NBF) for subsequent histological procedures.

### 2.2. Neurobehavioral studies

#### 2.2.1. Tail Suspension Test

The Tail Suspension Test (TST) was carried out as earlier described (Ijomone et al., 2015). The TST is widely used to evaluate despair-like or depressive behavior in rats. The task is based on the fact that rats develop an immovable posture after exposure to a short duration of inescapable stress of being suspended by their tail. Here, the TST apparatus consists of a wooden box (54×30×52 cm) with a hook in the center of the top side. The amount of time spent being immobile is recorded for each rat suspended individually by their tail from the hook with adhesive tape for 6 min.

#### 2.2.2. Y-maze Test

The Y-maze Test was carried out as earlier described (Ijomone et al., 2015). This test evaluates short-term spatial memory as an indicator of cognitive abilities using the spontaneous alternation behaviors of rats. Rats are placed in a start arm of the maze, which is Y-shaped. The rats are allowed to move freely for 8 minutes. Hind paws of the rats should be entirely within an arm to be considered as rats having entered the arm. When the rats enter all 3 arms in the overlapping triplet sets, it is defined as spontaneous alternation. Spontaneous alternation is calculated as a percentage of the number of alternations recorded using this formula;

[spontaneous alternation/(total number of arm entries−2)]×100.

### 2.3. Histological examinations

The histology of the hippocampus was assessed in brains excised from experimental rats. Brain tissues were processed for routine paraffin wax embedding. Then, 6-μm thin sections were obtained using a rotary microtome and stained using routine Hematoxylin and Eosin (H & E) for histological evaluation of neurodegeneration in the hippocampal region. The stained sections were examined under a digital microscope, and photomicrographs were obtained with the aid of an attached camera.

### 2.4. Statistical analysis

The obtained data were analyzed by 1-way ANOVA, followed by multiple comparison tests with Student Newman-Keuls (SNK). Additionally, the data were analyzed by 2-way ANOVA for gender and dose interactions. The results were expressed as mean±SEM, while statistical significance was set at P <0.05. All data analyses were done in statistical software GraphPad Prism (Version 5.03, GraphPad Software, USA) that plotted the dot-plot representation of data, too.

## 3. Results

### 3.1. DM induces depressive-like behavior

**Figure 1.**
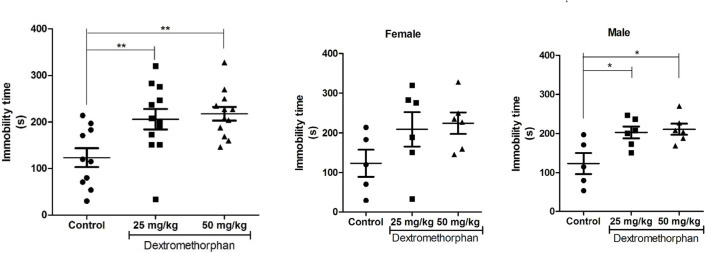
Effects of Dextromethorphan (DM) on depressive-like behavior in TST Top. Result obtained irrespective of sex (n =10/control, n=12/DM-treated groups); Bottom. Result obtained for female and male rats (n=5/control/sex, n=6/DM-treated groups/sex); * P<0.05; ** P <0.01

Results of TST showed that DM administration significantly increased immobility time irrespective of sex compared to that in the control (F_2,31_=6.67; P <0.01; 1-way ANOVA; Figure 1). However, when two sexes were compared separately, DM administration to female rats showed no significant effect, while administered-DM male rats exhibited significant increase in immobility when compared to respective control (F_2,14_=6.37; P<0.05; 1-way ANOVA; Figure 1). Additionally, 2-way ANOVA showed no significant interaction (F_2,28_=0.03; P=0.9744) between sex and dose following DM treatment, as well as no significant sex factor (F_2,28_=0.08; P=0.7826). However, there was a significant dose factor (F_1,28_=6.06; P<0.01).

### 3.2. DM induces cognitive deficits

**Figure 2:**
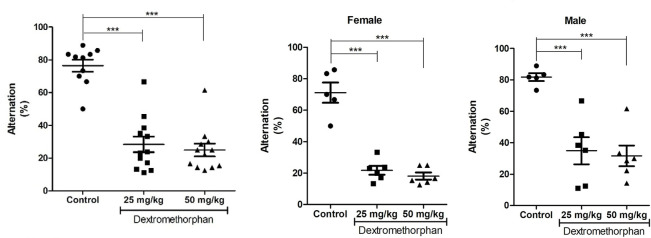
Effects of Dextromethorphan (DM) on cognitive behavior in Y-maze Top. Result obtained irrespective of sex (n=10/control, n=12/DM-treated groups); Bottom. Result obtained for female and male rats (n=5/control/sex, n=6/DM-treated groups/sex). *** P<0.001.

Analysis of data from the Y-maze test showed a significant reduction in the percentage of spontaneous alternation following DM administration irrespective of sex (F_2,31_=44.08; P<0.001; 1-way ANOVA; Figure 2). Comparing the results of sexes separately, both female (F_2,14_=53.51; P<0.001; 1-way ANOVA; Figure 2) and male (F_2,14_=16.06; P<0.001; 1-way ANOVA; Figure 2) rats showed a significant decrease in the percentage of alternation compared to their respective controls. Additionally, 2-way ANOVA showed no significant interaction between sex and dose following DM treatment (F_2,28_=0.04; P =0.9619). However, there was a significant dose factor (F_2,28_=50.87; P<0.001) and sex factor (F_1,28_=7.51; P<0.05).

### 3.3. DM administration produced no histological alterations in the hippocampus

**Figure 3.**
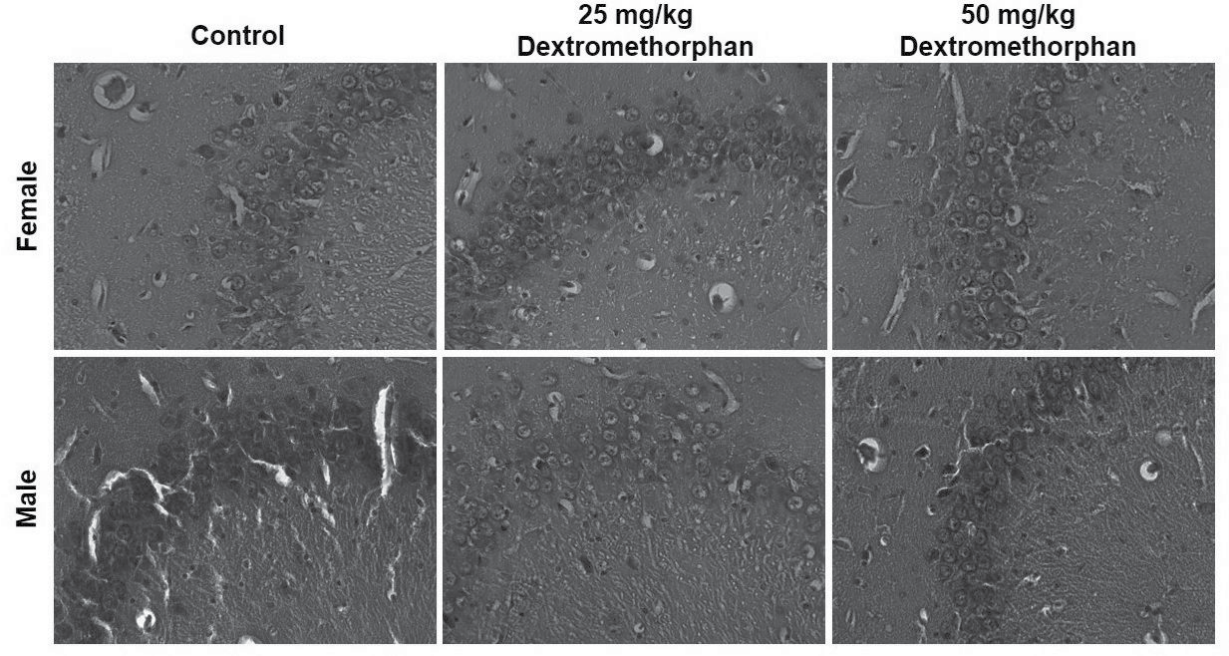
Representative photomicrographs of hippocampal CA fields (CA3) of control and dextromethorphan-treated rats H & E ×400. No histological alterations are observed following dextromethorphan treatment.

Histological observations of the hippocampus showed normal appearance following DM treatment, as seen in the control. Large pyramidal with conspicuous nucleoli cells of the hippocampal CA fields are observed and appeared intact (Figure 3).

## 4. Discussion

The present study demonstrates the depressive-like effect of DM. Also, we validate the cognitive impairment effect of DM without a change in the morphology of the hippocampus.

Although DM has been shown to produce rapid antidepressant activity in various mice studies (Nguyen et al., 2016; Nguyen & Matsumoto, 2015), according to our research, DM induces a depression-like state in the DM-treated rats, notwithstanding treatment dose or gender of rats. The state of depression in animals was assessed by the TST. Increased immobility time in the TST is the predictor of depression in rodents (Ijomone et al., 2015). While we cannot ascertain the reason for the DM-induced depressive state in rats, our finding agrees with the preclinical report of Po et al. (2015). They already showed that depression-like state was induced in male Sprague Dawley rats via attenuation of hippocampal neurogenesis (following 40 mg/kg IP of DM for 14 days). The state of depression was assessed in the TST and FST behavioral models.

Interestingly in our present study, only male rats showed depressive-like effects when female and male rats were analyzed separately. Considering that the above report of Po et al. (2015) that also used male rats, we posit that males may be more prone to DM-induced depressive-like behaviors. However, recent Swiss Webster mice studies findings are in contrast with the results of the present study (Nguyen et al., 2016; Nguyen & Matsumoto, 2015). These authors reported that DM reduced immobility time in the FST and TST, indicating the antidepressant effect.

Various receptor activities have been implicated in the rapid antidepressant effect of DM. One of such receptors is 5-HT1B/D receptors, leading to increased 5-HT levels as well as the modulation of norepinephrine reuptake (Codd, Shank, Schupsky, & Raffa, 1995). Also, the contribution of sigma-1 receptor agonist activity of DM has been demonstrated in its antidepressant effect in mice (Nguyen, Robson, Healy, Scandinaro, & Matsumoto, 2014). Sigma-1 receptor is a novel target for the potential new class of antidepressant drugs, partly due to its control of the expression levels and receptor regulation of α-amino-3-hydroxy-5-methyl-4-isoxazolepropionic acid (AMPA) (Nguyen & Matsumoto, 2015). However, the exact mechanism of action involving AMPA in the antidepressant effect of DM is still elusive, as AMPA receptors do not interact directly with DM (Werling, Keller, Frank, Nuwayhid, 2007).

Taken together, though DM was administered via intraperitoneal route and at the proper dosage, the mice studies suggest an antidepressant effect of DM while studies in rats (including the present study) demonstrate the depression-like effect of DM. It seems that the major difference between the rat and mice studies is the duration of the study. That is in the rat studies, DM was administered in repeated doses over days while in the mice studies, DM was applied only on the day of behavioral assessment. Whether DM induces a rapid antidepressant effect in animal models on the first day of administration and produces depressive-like state following repeated administration is yet to be determined.

In the current study, as assessed using the Y-maze, DM treatment compromised cognitive functions in both male and female rats compared to their respective controls. Spontaneous alternation, as obtained in the Y-maze test, is a classically used method to measure spatial working or short-term memory.

The decrease in percentage alternation is indicative of compromised spatial short-term memory (Ijomone et al., 2015). This finding supports previous reports that DM impairs short-term cognitive functions assessed by other behavioral models, including the passive avoidance test (Murata & Kwasaki, 1993) and Morris water maze test (Zhang et al., 2007; Bane et al., 1996). Similarly, in humans, 120 mg DM does not alter long-term memory in drivers (Perry et al., 2015).

DM at 100–300 mg/70 kg weight creates acute impairments in the working memory of adults (Carter et al., 2013). Moreover, Bane et al. (1996) revealed that DM impairment of cognitive functions was dose-dependent. Nevertheless, our observation is at variance with their reports, as there were no differences in the mean percentage alternation between the groups that received 50 mg/kg and 25 mg/kg and regardless of gender.

The mice study of Zarrindast et al. (2014) suggests that DM alters cognitive functions due to blocking NMDA receptors in the dorsal hippocampus since the systemic and intra-cerebral injection of DM in the hippocampal region show similar findings on cognitive impairment. Correspondingly, other studies have shown that other NMDA-receptor antagonists may decrease memory retention and alter spatial discrimination during learning tasks (Watson & Stanton, 2009; McHugh et al., 2008). Hence, hippocampal NMDA receptors are crucial in the processes of learning and memory (Berg et al., 2013).

The hippocampus is known for its significant role in cognition (Ijomone, Nwoha, Olaibi, Obi, & Alese, 2012; Squire, 2009) and depressive behaviors (Balfour & Ridley, 2000). Interestingly, this study did not detect any observable histologic alterations in the CA areas of the hippocampal regions of both male and female rats at the given doses of DM. Acute administration of DM at the given doses is not debilitating enough to manifest deficits in the hippocampal morphology, even though it results in behavioral impairments associated with hippocampal functions. Similarly, Carliss et al. (2007) did not find any morphologic changes in the brain of rats following oral gavage of DM in rats. Though, it is noteworthy that cognitive and depressive behaviors are heavily influenced by other brain regions such as amygdala (Zald, Hagen, & Pardo, 2002), nucleus accumbens (Bar-Haim et al., 2009), basal ganglia, thalamus, frontal lobe (including gyrus rectus and orbitofrontal cortex) (Kempton et al., 2011; Arnone et al., 2011), and the anterior cingulate gyrus (Drevets, Savitz, & Trimble, 2008).

To the best of our knowledge, this is the first report to investigate the dimorphic sexual effect of DM with regard to cognitive functions and depression in rats. In conclusion, IP administration of DM at 50 mg/kg and 25mg/kg may induce depression-like state and impair short-term memory in both male and female rats without histologic defects in the hippocampus. Also, our data suggest that the effects of DM on depressive behaviors may be influenced by gender.
